# Neuroglobin promotes neurogenesis through Wnt signaling pathway

**DOI:** 10.1038/s41419-018-1007-x

**Published:** 2018-09-20

**Authors:** Zhanyang Yu, Chongjie Cheng, Yu Liu, Ning Liu, Eng H. Lo, Xiaoying Wang

**Affiliations:** 1 Neuroprotection Research Laboratory, Departments of Neurology and Radiology, Massachusetts General Hospital, Harvard Medical School, Charlestown, MA USA; 2 Department of Neurosurgery, The First Affiliated Hospital of Chongqing Medical University, Chongqing, China; 3 Department of Neurology, the Second Affiliated Hospital, Harbin Medical University, Harbin, Heilongjiang China; 4 College of Medicine, Hunan Normal University, Changsha, China

## Abstract

Neuroglobin (Ngb) has been demonstrated by our lab and others to be neuroprotective against neurological disorders including stroke. However, the roles of Ngb in neurogenesis remain elusive. Neurogenesis can occur in adulthood and can be induced by pathological conditions in the brain such as stroke, and significantly contributes to functional recovery, thus enhancing endogenous neurogenesis may be a promising therapeutic strategy for neurodegenerative diseases. In this study we aimed to investigate the roles of Ngb in neurogenesis using Lentivirus overexpressing Ngb (Lv-Ngb). We show that Ngb overexpression promoted the proliferation of neural progenitor cells (NPC) marked by increased neurosphere number and size. Ngb overexpression also enhanced neuronal differentiation of cultured NPC under differentiation conditions. Moreover, subventricular injection of Lv-Ngb in mice after middle cerebral artery occlusion (MCAO) increased PSA-NCAM positive neuroblasts and Tuj1 positive immature neurons, suggesting that Ngb overexpression promotes neurogenesis in mice brain after stroke. We further show that the pro-neurogenesis effect of Ngb overexpression might be mediated through Dvl1 up-regulation, and subsequent activation of Wnt signaling, indicated by increased nuclear localization of beta-catenin. These results suggest that Ngb may play an important role in promoting neurogenesis in neurodegenerative diseases such as stroke, which may eventually benefit the development of stroke therapeutics targeting neurogenesis through Ngb upregulation.

## Introduction

Neuroglobin (Ngb) is an oxygen-binding globin protein predominantly expressed in brain neurons. Since its discovery in 2000^1^, a large body of evidence by our lab and others have demonstrated that Ngb is an endogenous neuroprotective molecule against neurodegenerative diseases including stroke and brain trauma^2–5^. Enhanced Ngb gene expression reduces the histological and functional deficits after stroke^6–9^. Conversely, Ngb knock-down deteriorates the outcome of hypoxic/ischemic brain injury^10^. Ngb has been demonstrated to be a molecular sensor for hypoxia-induced oxidative stress and a scavenger of free radicals^4,11^. Ngb may also function as a guanine nucleotide dissociation inhibitor (GDI), inhibiting GDP dissociation from Gα subunit of G protein and therefore reducing cell death^12^. Additionally, Ngb may exert its neuroprotective effect through preserving mitochondrial function and intervening apoptosis pathways via interaction with mitochondrial proteins VDAC and Cyt-c^3,13,14^. The neuroprotective effect of Ngb is not limited to stroke. For example, Ngb overexpression is also protective against beta-amyloid induced neurotoxicity and Alzheimer’s phenotypes^15^. Clinical studies revealed that Ngb protein level is decreased during aging^16^, implying a role of Ngb in aging-related neurodegeneration. These findings support that Ngb is a promising therapeutic target for promoting endogenous neuroprotection.

Despite the well established neuroprotective function of Ngb, whether it is also involved in neurogenesis has not been clarified. Neurogenesis is a process of generating functional neurons from precursor cells. It has been widely recognized that neurogenesis can occur not only in embryonic and perinatal stages, but also in adulthood of mammals^17^. Adult neurogenesis is a complex process consisting of proliferation, differentiation, migration, survival and functional integration of newly formed neurons to existing neuronal circuits^17^. The two prominent regions of adult neurogenesis are the subventricular zone (SVZ)^18–20^ and subgranular zone (SGZ) of dentate gyrus (DG)^21^. Studies using conditional NPC ablation technique confirmed that neurogenesis following brain injury such as stroke significantly contributes to the brain function recovery^22^. Exogenous stem cell therapies, namely transplantation of induced pluripotent stem cells (iPSC), mesenchymal stem cells or embryonic stem cells, have been proved to be beneficial for brain recovery in animal stroke studies^23,24^, however they are still far from clinical practice due to adverse side effects such as tumor formation and immune-reaction^25^. These findings support that promoting endogenous neurogenesis might be a more promising strategy for treatment of neurological diseases.

Interestingly, a number of studies imply a potential role of Ngb in neurogenesis. Ngb has been reported to be expressed early in the course of neuronal differentiation^26^. Moreover, mitochondria play a key role in neurogenesis by providing ATP as the energy for cytoskeleton remodeling and neurite outgrowth^27^. Our lab has demonstrated that Ngb preserves mitochondrial ATP production^8^, and is physically localized inside mitochondria^28,29^, therefore Ngb may engage in neurogenesis via improving mitochondrial function. Importantly, adult neurogenesis can be induced under pathological conditions such as stroke^18,30^, which is in line with experimental studies that mild hypoxia^31,32^, oxidative stress^33^ and HIF-1^34^ can stimulate neurogenesis and neuronal differentiation. Since Ngb is inducible by both hypoxia^6^ and HIF-1^35^, and acts as a sensor for oxidative stress^36,37^, these findings strongly suggest a potential role of Ngb in neural progenitor cell (NPCs) proliferation and neuronal differentiation in neurological disorders. In this study we therefore aim to investigate the roles and mechanisms of Ngb in neurogenesis using Ngb overexpression in cultured NPC, and further validate it in mice stroke models. As we have established a cell based screening system for identifying compounds that upregulates endogenous Ngb expression^38^, this study may benefit the development of stroke treatment targeting neurogenesis through Ngb upregulation.

## Materials and methods

### Animals

All animal experiments were performed following protocols approved by the Massachusetts General Hospital Institutional Animal Care and Use Committee in compliance with the NIH Guide for the Care and Use of Laboratory Animals.

### Neural progenitor cell culture

SVZ neural progenitor cells were isolated from normal mice at age of 10–11 weeks and dissociated with mechanical trituration. The cells were then plated at a density of 2 × 10^4^ cells/ml in Dulbecco’s modified Eagle’s Medium-F12 (DMEM/F12) (Invitrogen) containing L-glutamine (2 mM), glucose (0.6%), putrescine (9.6 μg/ml), insulin (0.025 mg/ml), progesterone (6.3 ng/ml), apo-transferrin (0.1 mg/ml), and sodium selenite (5.2 ng/ml), 20 ng/ml of epidermal growth factor (EGF) (R&D Systems, Minneapolis, MN), and 20 ng/ml of basic fibroblast growth factor (bFGF) (R&D Systems). After incubation at 37 °C for a few days, neurospheres were generated (primary sphere), and passaged by mechanical dissociation and reseeded at a density of 20 cells/μl in bFGF- and EGF-containing media (passage 1 cells). The cells were then used for various experiments. These cells were cultured in reduced growth medium containing 10 ng/ml of EGF and 10 ng/ml of bFGF.

To analyze the formation of secondary neurospheres, passage 1 neurospheres were collected and digested with 0.05% trypsin-EDTA (Invitrogen) for 5 min at 37 °C. They were then gently triturated with a fire-narrowed Pasteur pipette, spun down at 400 rpm for 3 min, re-suspended in the reduced growth medium, and plated at 1 × 10^4^cells/ml in each well of a 24-well plate (Corning). The number of neurospheres was counted at 7 days in vitro.

To analyze NPC proliferation, bromodeoxyuridine (BrdU) (20 μg/ml), the thymidine analog that is incorporated into the DNA of dividing cells, was added into culture medium 18 h before the cells were examined by immunostaining using anti-BrdU antibody.

To study the differentiation of NPC, neurospheres were mechanically dissociated to single cells, and then plated (2.5 × 10^4^ cells/cm^2^) onto laminin-coated glass coverslips in differentiation medium, which is DMEM/F12 containing 2% fetal bovine serum (FBS) but without the growth factors, for 7 days. Immunocytochemistry was performed with various antibodies (see below) to determine the phenotypes of NPC differentiation.

### Lentivirus packaging

Mouse Ngb cDNA sequence was cloned into parental vector pCDH-MSCV-MCS-EF1-Puro (System Biosciences, Palo Alto, CA) between the Xba I and BamH I sites. Control vector pCDH-MSCV-MCS-EF1-GFP containing GFP gene was also purchased from System Biosciences. Viral packaging and titer determination were performed by MGH Viral Core Facility (Charlestown, MA, USA).

### Lentivirus transduction into NPC

To transfect the cultured NPCs with the lentiviral particles, 50 μl viral solution (1 × 10^6^ TU/ml) were added to proliferating NPC in 6-well dishes, incubated for 24 h, and then washed 3 times to stop the infection. The expression of the infected genes was confirmed by mCherry or GFP expression by fluorescence microscopy and Western blot analysis.

### Immunocytochemistry

Immunocytochemistry was performed as we previously described^39^. Briefly, cultured neurospheres or differentiated NPCs were washed with cold PBS (pH 7.4), and fixed with 4% paraformaldehyde (PFA) for 30 min. The cells were then washed with cold PBS containing 0.1% Tween for 4 times, 5 min each wash, and further blocked with 5% FBS for 1 h. Next the cells were incubated with the following primary antibodies: anti-Nestin (1:300, Abcam), anti-doublecortin (Dcx) (1:300, Abcam), mouse anti-BrdU (1:100; Roche Applied Science), mouse anti-β-tubulin III (Tuj-1, 1:500; Covance), rabbit anti-β-catenin (1:1000, Abcam), rabbit anti-glial fibrillary acidic protein (GFAP) (1:500; Invitrogen), mouse anti-PSA-NCAM (1:200, ThermoFisher). After PBS washing, the cells were then incubated with the primary antibodies listed above and with Cy3- or fluorescein isothiocyanate (FITC)-conjugated secondary antibodies. Vectashield (Vector Laboratory, Burlingame, CA) was used to coverslip the immunocytochemic slides. Immunostaining was analyzed using a fluorescence microscope (Olympus BX51). Quantification of neurite length in differentiated immature neurons (Tuj-1 positive) was performed using ImageJ-Neurite Tracer.

### Nulcear protein isolation

Nuclear extract was prepared from cultured NPCs or mouse brain tissue using NE-PER Nuclear and Cytoplasmic Extraction Reagents (Pierce) following the manufacturer’s instructions. Briefly, for cultured cells, the cells were collected and washed with PBS, then re-suspended in cytoplasmic extraction regents I (CER I). For brain tissue, the tissue was homogenized in CER I using Dounce Homogenizer. The samples were then vigorously vortexed for 15 s followed by incubation on ice for 10 min. Then cytoplasmic extraction regents II (CER II) was added to the tube, vortexed for 5 s, and incubated on ice for 1 min. The tube was vortexed again for 5 s, followed by centrifugation at highest speed (16,000 × *g*) for 5 min. Then the supernatant (cytoplasmic extract) was transferred to another tube. The pellet (containing nuclear extract) was suspended in appropriate volume of nuclear-extract reagent (NER), vortexed for 15 s every 10 min on ice, for total 40 min. Then the tube was centrifuged at 16,000 × *g* for 10 min, and the supernatant (nuclear extract) was transferred to a new tube for further analysis.

### Western blot

Western blot was performed as we previously described^39^. Briefly, total protein extract was prepared by homogenizing cultured NPC or mice brain tissue using lysis buffer. The concentration of either total protein or isolated nuclear protein was measured with Bradford reagent (Bio-Rad laboratories, Hercules, CA), and 30–50 μg protein from each sample was loaded onto a SDS gel for electrophoresis and transferred to nitrocellulose membranes. The blots were reacted with the following primary antibodies: chicken anti-Ngb (1:2000; BioVendor, Candler, NC), mouse anti-Dvl1 (1:1000, Santa Cruz), rabbit anti-β-catenin (1:1000, Abcam), mouse anti-Histone-H3 (1:1000, cell signaling), mouse anti-β-actin (1:5000, Millipore, Billerica, MA) as a control in a solution containing 0.05% Tween-20, 1% bovine serum albumin, and 4% nonfat dry milk at 4 °C overnight. The membrane was incubated with the corresponding horseradish peroxidase-conjugated secondary antibody (1:2000) for 1 h at room temperature. An enhanced chemiluminescence system (Pierce; Thermo Fisher Scientific) was used for antibody detection.

### Transient cerebral focal ischemia

Anesthesia was induced with 1.5% isoflurane in a mixture of 70% nitrous oxide and 30% oxygen delivered by face mask. Focal cerebral ischemia was induced by introducing a silicone-coated 6–0 monofilament until it occluded the origin of the right MCA. The rectal temperature was maintained at 37 ± 0.5 °C and the regional cerebral blood flow of the right front parietal cortex was continually monitored during the surgical procedure. The filament was withdrawn after 45 min to allow reperfusion of the ischemic hemisphere. The operator was blinded to the treatment status of the animals.

### Introcerebroventricular (ICV) injection of lentivirus in mice

To administer the virus, the mice were anesthetized by 1.5% isoflurane and positioned on a stereotactic frame (SR-6N; Narishige, Tokyo, Japan). The skin over the skull was incised, and a small hole was made in the skull above the target using a microdrill. The stereotactic coordinates were as following: anteroposterior (AP), −0.4 mm; mediolateral (ML), −1.0 mm; dorsoventral (DV), −3.0 mm from the bregma for injection into the right lateral ventricle. The mice were injected unilaterally with 6 μl lentivirus solution (1 × 10^7^ TU/ml) using a 10 μl Hamilton syringe with a 0.52 mm needle (Hamilton Co., Reno, NV). The virus was injected over 10 min, and the needle was left in place for 5 min prior to withdrawal.

### Immunohistochemistry

Before being sacrificed, mice were treated with anesthetized by 1.5% isoflurane and cardio-perfused with saline. The brain was dissected and sectioned using cryostat, sections being stored at -80 °C. Before immunohistochemistry, brain slices were air dried and fixed in 4% paraformaldehyde, then blocked in 5% fetal bovine serum for 80 min. After incubation overnight at 4 °C with rat anti-mouse PSA-NCAM (1:100, ThermoFisher), anti-Tuj1 (1:100, Covance), the sections were washed with PBS, and then incubated with FITC- or TRITC-labeled secondary antibodies at room temperature for 1 h. After washing with PBS, slides were cover-slipped with Crystal mount (Biomeda), and analyzed using fluorescence microscope (ECLIPSE Ti-s, Nikon). For quantification of PSA-NCAM and Tuj1 positive cells in mice after stroke, the Area of Interest (AOI) is peri-infarct brain region including cortex, hippocampus and SVZ. Five sections per mice were digitized using a ×20 objective, and the PSA-NCAM and Tuj1 positive cells were counted in a blinded way.

### Statistical analysis

All data were expressed as mean ± SD. The number of samples was 6 per group for mice stroke models and 6 per group for in vitro experiments. The power calculation was performed using information collected from a preliminary study that was conducted under the same conditions. Multiple comparisons were evaluated by one-way ANOVA followed by Tukey–Kramer’s tests. For all comparisons, *p* < 0.05 was considered statistically significant.

## Results

### Cultured neurospheres have phenotypes of neural progenitor cells (NPC)

**Fig. 1:**
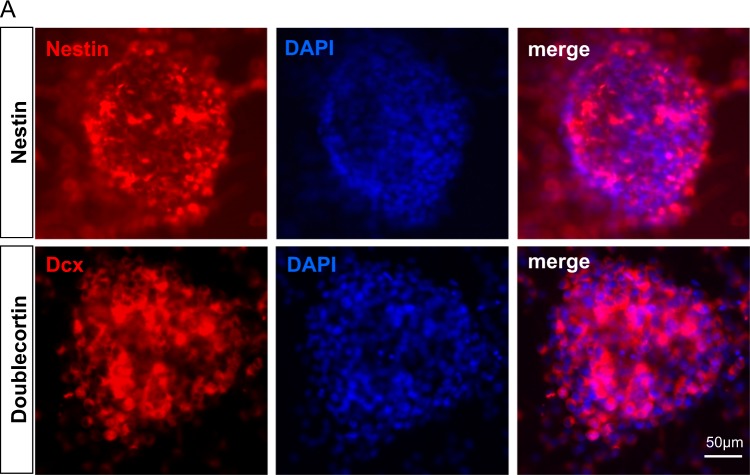
Cultured neurospheres have phenotypes of neural progenitor cells. Neurosphere was cultured from isolated adult mouse SVZ region. Neurospheres were formed after 7 days. Neurosphere phenotype was examined by immunostaining for neural progenitor cell marker nestin and doublecortin

NPC isolated from the SVZ of adult mouse were plated at a density of 20 cells/μl in DMEM/F12 containing progesterone, insulin, transferrin, bFGF and EGF. Neurospheres were formed after 7 days in vitro. We then performed immunocytochemistry for the neurospheres using antibodies against nestin and doublecortin (Dcx), the NPC markers. Our results show that the majority of cells in neurospheres were nestin- and Dcx-immunoreactive (Fig. 1), indicating that the cultured neurospheres have phenotypes of neural progenitor cells.

### Ngb overexpression promotes the proliferation of cultured NPC

**Fig. 2:**
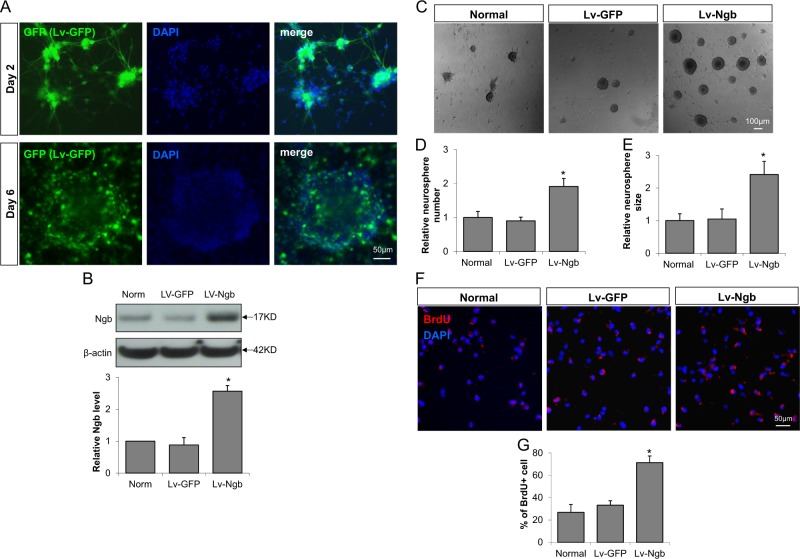
Ngb overexpression promotes NPC proliferation. Lentivirus containing Ngb (Lv-Ngb) or GFP (Lv-GFP) were transduced to neurosphere at day 2 of culture. NPC proliferation was examined by measuring neurosphere number and size, and BrdU corporation 6 days later. **a** Lv-GFP transduction into neurosphere and microscopic imaging of GFP; **b** The efficiency of Lv-Ngb transduction was tested by measuring Ngb protein level in neurosphere using Western blot. (*n* = 4, * *p* < 0.05vs Lv-GFP). **c** Representative images of cultured neurosphere; **d** Quantification of relative neurosphere number, **e** Quantification of relative neurosphere size (*n* = 6, *p < 0.05 vs Lv-GFP); **f** Representative images of BrdU immunostaining; **g** Quantification of the percentage of BrdU positive cell (*n* = 4, *p < 0.05 vs Lv-GFP)

To investigate the roles of Ngb in neurogenesis, we first tested the effect of Ngb-overexpression on the proliferation of cultured NPCs. Ngb overexpression was achieved through transduction of Lentivirus containing Ngb gene (Lv-Ngb) at day 2 of neurosphere culture. Lentivirus containing GFP (Lv-GFP) was used as control. We show that the lentivirus was successfully transduced into cultured NPCs indicated by GFP expression (Fig. 2a). Western blot showed that Ngb protein level in Lv-Ngb group was significantly increased compared to normal or Lv-GFP group (Fig. 2b).

We next found that, at 7 days in NPC culture, the number and size of Lv-Ngb transduced neurospheres were significantly higher than normal or Lv-GFP groups (Fig. 2c–e). To further study the effect of Ngb overexpression on NPC proliferation, we added BrdU in culture medium and measured BrdU incorporation 18 h later by immunostaining. Lv-Ngb transduction significantly increased the number of BrdU positive cells compared to normal or Lv-GFP transduced cells (Fig. 2f, g). These data suggest that Ngb-overexpression promotes NPC proliferation.

### Ngb overexpression promotes neuronal differentiation of cultured NPC

**Fig. 3:**
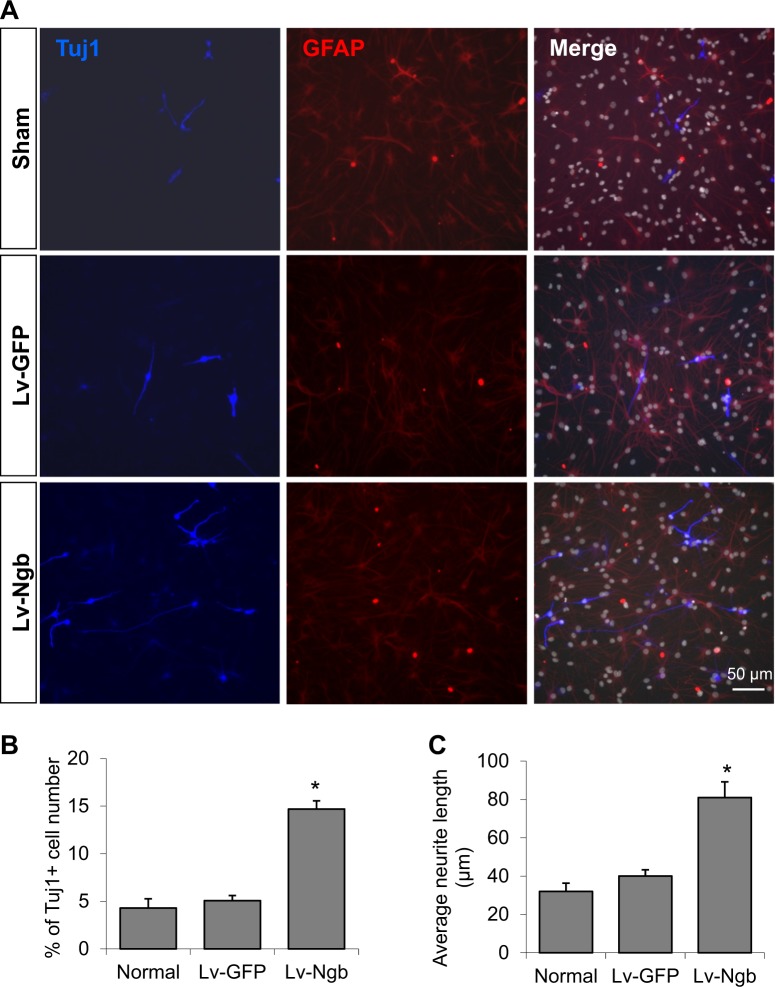
Ngb overexpression promotes neuronal differentiation of cultured NPC. NPC was cultured in differentiation medium (DMEM/F12 + 2%FBS) and Lentivirus containing Ngb or GFP gene was transduced on day 2 of culture. Differentiated cells were examined by immunocytochemistry using anti-Tuj1 (immature neuron maker) and anti-GFAP antibodies. NPC differentiation was examined by immunostaining for Tuj1 (immature neuron) and GFAP (astrocytes). **a** Representative images of Tuj1 and GFAP immunostaining; **b** Quantification of percentages Tuj1 positive cells, **c** Quantification of average neurite length (*n* = 4,* *p* < 0.05 vs Lv-GFP)

We next investigated the effect of Ngb overexpression on NPC differentiation. Cultured neuroshperes were dissociated into single cells and plated in differentiation medium (DMEM/F12 + 2%FBS). Lentivirus was transduced on day 2 of culture, and differentiated cells were examined by immunocytochemistry using anti-Tuj1 (immature neuron maker) and anti-GFAP (astrocyte marker) antibodies. We show that the percentage of Tuj1 positive cells in Lv-Ngb transduced NPCs was significantly higher than control groups (Fig. 3a, b). Furthermore, the neurite length of Tuj1 positive cells was quantified with ImageJ-Neurite Tracer. The average neurite length of newly differentiated neurons in Lv-Ngb transduced NPCs was significantly increased compared to control groups (Fig. 3c). These data suggest that Ngb overexpression may enhance neuronal differentiation of cultured NPC.

### Wnt signaling regulates the effect of Ngb in promoting NPC proliferation and neuronal differentiation

**Fig. 4:**
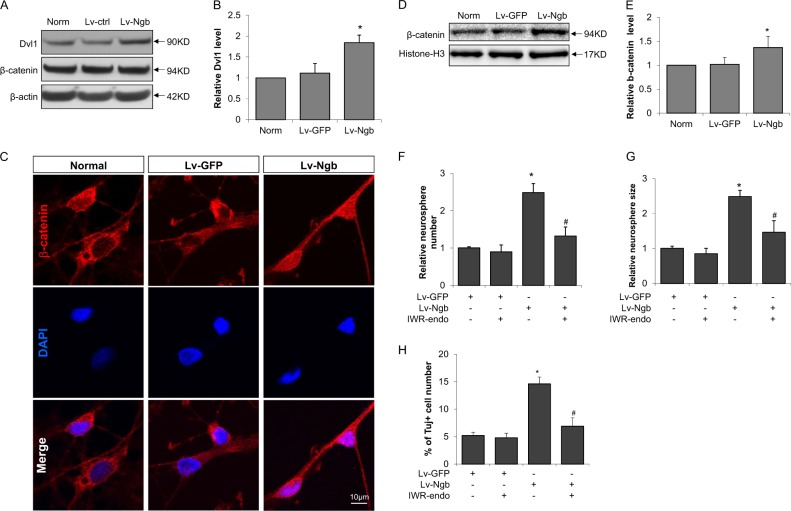
Wnt signaling is involved in the promotional effect of Ngb overexpression on neurogenesis. To investigate the mechanisms of the neurogenesis promotional effect by Ngb overexpression, we examined the protein levels of Dvl1 and β-catenin, the two key components of Wnt signaling, after Lv-Ngb transduction in cultured neurosphere. **a** Representative Western blot images for Dvl1 and β-catenin using cell lysate of cultured NPCs. β-actin was used as loading control. **b** Quantification of Dvl1 protein level; **c** Immunostaining for β-catenin in cultured NPC; **d** Representative Western blot images and **e** quantification of β-catenin in isolated nuclear proteins. Histone-H3 was used as nucleus loading control (*n* = 4,* *p* < 0.05 vs Lv-GFP). We further used a Wnt inhibitor, IWR-endo, to test the involvement of Wnt signaling in the neurogenesis promotional effect by Ngb overexpression; **f** Relative neurosphere number after Lv-Ngb transduction and IWR-endo treatment; **g** Relative neurosphere size after Lv-Ngb transduction and IWR-endo treatment; **h** The percentage of Tuj positive cell numbers in NPC differentiation after Lv-Ngb transduction and IWR-endo treatment (*n* = 4, *p < 0.05 vs Lv-GFP, ^#^*p* < 0.05 vs Lv-Ngb)

Wnt signaling involving Dvl1 and β-catenin is an important pathway that regulates adult neurogenesis^40,41^. To dissect the potential mechanisms of the effect of Ngb overexpression on neurogenesis, we investigated the roles of Wnt signaling in this process. We first investigated the effect of Ngb overexpression on the expression of Wnt signaling components. We show that Ngb overexpression significantly increased Dvl1 protein level in cultured NPCs compared to control, whereas the total protein level of β-catenin, a key component of canonical Wnt signaling^41^, was not obviously changed (Fig. 4a, b). Since β-catenin first accumulates in cytoplasm and subsequently translocates to nucleus to function as a transcriptional coactivator of Wnt target genes^42^, we further examined the nuclear localization of β-catenin in cultured NPCs. Ngb overexpression significantly increased the nuclear distribution of β-catenin by both immunocytochemistry (Fig. 4c) and Western blot for isolated nuclear proteins (Fig. 4d, e), suggesting that Ngb may affect Wnt signaling by regulating the nuclear translocation of β-catenin.

To further define the roles of Wnt signaling in the promotional effect of Ngb on neurogenesis, we used a Wnt inhibitor, IWR-endo^43^, to treat cultured NPCs. We show that IWG-endo treatment partially reversed the promotional effect of Ngb overexpression on both neurosphere number and size (Fig. 4f, g). Furthermore, for NPCs under differentiation conditions, IWG-endo also partially but significantly blocked the promotional effect of Ngb on neuronal differentiation marked my Tuj1 immunostaining (Fig. 4h). These data suggest that the effect of Ngb overexpression on neurogenesis is at least in part via Wnt signaling.

### Ngb overexpression enhances neurogenesis after transient focal ischemic stroke in mice

**Fig. 5:**
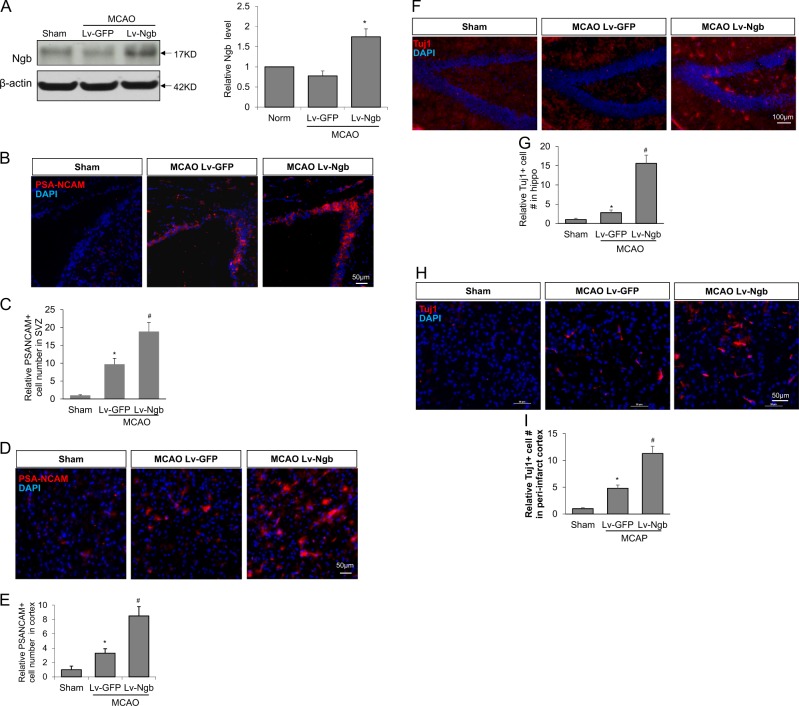
Ngb overexpression improved neurogenesis in mice after MCAO. To test the effect of Ngb overexpression on neurogenesis after stroke, we performed transient MCAO in mouse and administered Lv-Ngb or Lv-GFP through ICV injection at 2 days after stroke. **a** Ngb protein level was measured by Western blot to confirm Ngb overexpression by Lv-Ngb transduction at 5 days post stroke. NPC proliferation was assessed at 7 days post stroke using PSA-NCAM immunostaining. **b** PSA-NCAM immunostaining in SVZ area; **c** Quantification of PSA-NCAM positive cells in SVZ regions; **d** PSA-NCAM immunostaining in peri-infarct cortex region; **e** Quantification of PSA-NCAM positive cells in SVZ regions. Furthermore, differentiated immature neurons were assessed at 10 days post stroke using Tuj1 immunostaining. **f** Tuj1 immunostaining in hippocampus region; **g** Quantification of Tuj1 positive cells in hippocampus region; **h** Tuj1 immunostaining in peri-infarct cortex region; **i** Quantification of Tuj1 positive cells in peri-infarct cortex area. (*n* = 6,* *p* < 0.05 vs sham, ^#^*p* < 0.05 vs MCAO Lv-GFP)

To further validate the roles of Ngb in neurogenesis under pathological conditions, we examined whether Ngb overexpression may alter neurogenesis after ischemic stroke. We made transient MCAO (45 min) stroke models in mice. Intracrebroventricular (ICV) injection of Lv-Ngb or Lv-GFP was performed at 2 days after stroke. Western blot shows that ICV injection of Lv-Ngb significantly increased Ngb protein level in peri-infarct cortex at 3 days after injection (Fig. 5a). Moreover, GFP fluorescence imaging confirms the successful transduction of Lv-GFP (Fig. S1). At 7 days post MCAO, the mice were sacrificed and sectioned for immunostaining of neuroblast marker PSA-NCAM. MCAO significantly increased PSA-NCAM positive neuroblast numbers in SVZ and peri-infarct cortex compared to sham (MCAO Lv-GFP control group), whereas Ngb-overexpression further significantly increased the number of PSA-NCAM positive neuroblasts in SVZ (Fig. 5b, c) and in peri-infarct cortex (Fig. 5d, e) compared to MCAO Lv-GFP group.

Moreover, we performed immunohistochemistry for Tuj1 as a marker of newly generated immature neurons at 14 days post MCAO. MCAO significantly increased the number of Tuj1 positive cells in hippocampus and peri-infarct cortex compared to sham group, whereas Ngb-overexpression by Lv-Ngb further significantly increased the number of Tuj1 positive cells in hippocampus(Fig. 5f, g) and in peri-infarct cortex (Fig. 5h, i). These data indicate that Ngb overexpression may promote neurogenesis in mice after stroke.

### Effect of Ngb overexpression on Dvl1 and β-catenin protein levels after stroke

**Fig. 6:**
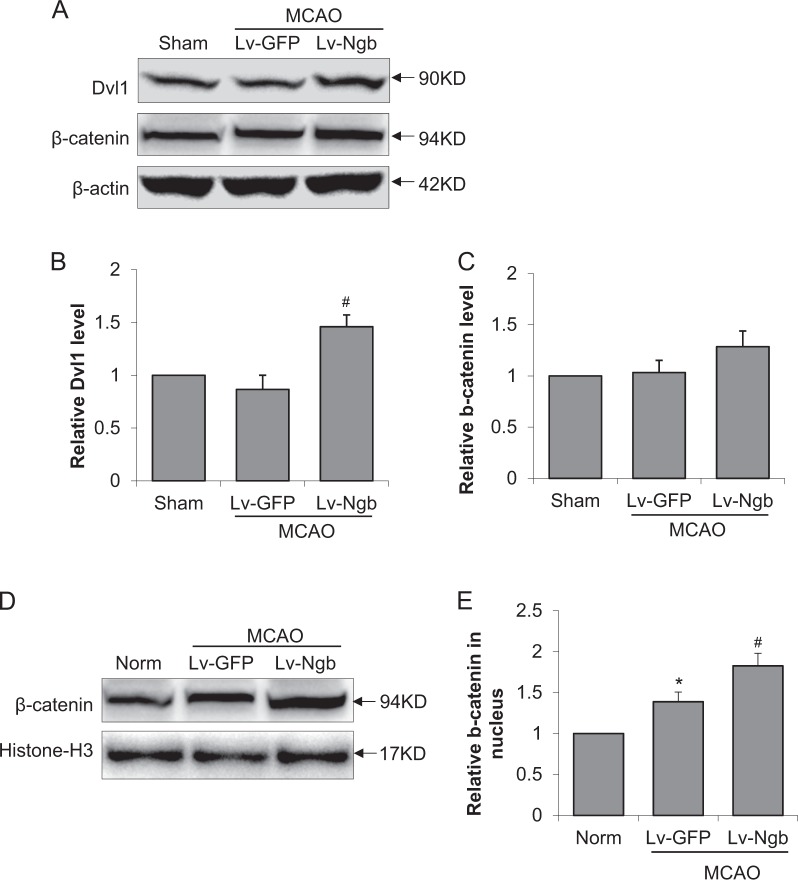
Effect of Ngb overexpression on Dvl1 and β-catenin expression after stroke. To further investigate the involvement of Wnt signaling in Ngb-overexpression-induced neurogenesis, we tested the protein levels of Dvl1 and β-catenin in peri-infarct mice brain at 5 days after stroke. **a** Representative images of Western blot for Dvl1 and β-catenin from peri-infarct brain lysate; **b**, **c** Quantification of Dvl1 and β-catenin; **d** Representative images of Western blot for β-catenin from isolated nuclear protein; **e** Quantification of β-catenin in nuclear extract. (*n* = 4, *p < 0.05 vs sham, ^#^*p* < 0.05 vs MCAO Lv-GFP)

To further investigate the involvement of Wnt signaling in Ngb-overexpression-induced neurogenesis, we tested the protein levels of Dvl1 and β-catenin in peri-infarct area of mice brain at 5 days after MCAO. Similar as in vitro data, for total tissue lysate, we found that Ngb overexpression significantly increased Dvl1 protein level compared to Lv-GFP group after MCAO, whereas the total protein levels of β-catenin was not obviously changed (Fig. 6 a–c). However, for nuclear extract from mice brain, Western blot shows that MCAO increases β-catenin level in nucleus compared to sham, whereas Ngb overexpression further significantly increased the nuclear β-catenin level compared to Lv-GFP group (Fig. 6 d, e). These data imply that Wnt signaling components Dvl1 and β-catenin might be at least partially involved in the promotional effect of Ngb overexpression on post-stroke neurogenesis.

## Discussion

In the present study, we found that Ngb overexpression promotes both proliferation and neuronal differentiation of cultured NPCs in vitro. Moreover, Ngb overexpression significantly enhanced the number of neuroblasts and newly differentiated immature neurons in mice after stroke. Mechanistically, we found that Ngb overexpression increased the levels of Dvl1, an effector of Wnt signaling pathway, and also enhanced the nuclear localization of β-catenin in both cultured NPC and mice brain after stroke. Moreover, the Wnt inhibitor significantly ameliorated the promotional effect of Ngb overexpression on NPC proliferation and neuronal differentiation. These data suggest that Ngb overexpression may promote neurogenesis after stroke, at least in part via Wnt signaling.

Recent experimental findings suggest that neuronal replacement by endogenous neural stem cells (NSCs) significantly contributes to the functional improvement after stroke^44,45^, which mainly occurs in SVZ and SGZ, and also in other brain regions along the ventricular system under pathological conditions^46^. In this study we observed significantly increased neuroblasts in SVZ after stroke, which confirms the stroke-induced neurogenesis. However, to achieve more neural repair for functional recovery after stroke, this endogenous neurogenesis needs to be further enhanced. In this study we for the first time demonstrated that Ngb-overexpression further enhances stroke-induced neurogenesis, putatively though Wnt signaling. As our lab and others have extensively documented that Ngb confers neuroprotection against stroke through ROS scavenging, improving mitochondrial function and suppressing apoptosis^2,47^, the findings in this current study will broaden our understanding of Ngb functions in stroke pathogenesis, and suggest that the enhanced neurogenesis may be an additional mechanism contributing to the improved neurological outcomes after stroke by Ngb overexpression^9^.

A large volume of studies have suggested that activation of endogenous neurprotection mechanisms might be a more promising strategy for stroke treatment. Our finding of Ngb promoting neurogenesis may open a new avenue for stroke treatment. We have established a cell based screening system and identified a few chemical compounds that up-regulate endogenous Ngb expression^38^, which may facilitate the development of stroke therapeutics targeting neurogenesis through Ngb upregulation. In this study we performed ICV injection of Lv-Ngb at 2 days and detected elevated Ngb protein level at 5 days after MCAO, which approximately corresponds to sub-acute phase of stroke. In future studies it may be necessary to try later time points to better mimic a treatment at recovery phase of stroke.

With the pro-neurogenesis role of Ngb overexpression being presented above, however, a very recent study using Ngb-knockout (Ngb-KO) NSC drew controversial conclusion, in that Ngb loss leads to increased growth and proliferation of cultured embryonic NSC^48^. A potential explanation for this contradiction is that inborn Ngb deficiency might induce compensatory mechanisms during development. Similar contradictions have been seen in the study by Hundahl et al^49^ showing that Ngb deficiency in mouse does not affect neuronal survival in severe hypoxia, which is also controversial to the big volume of evidence that Ngb overexpression protects against OGD/Hypoxia. In future studies we may use cell type specific and inducible Ngb gene knockout to further clarify these controversies.

Dvl1 is a member of the disheveled protein family that is involved in Wnt signaling pathways. Wnt signaling activates Dvl1, and triggers the stabilization of cytoplasmic β-catenin, which enters the nucleus and activates the transcription of Wnt target genes, thereby playing key roles in neurogenesis^50^. In this study we found that Ngb overexpression increased Dvl1 level and nuclei localization of β-catenin, suggesting enhanced Wnt signaling by Ngb overexpression. Moreover, Wnt inhibitor blocked the promotional effect of Ngb overexpression on proliferation and neuronal differentiation of cultured NPCs. In mice stroke models, stroke itself increased nuclei localization of β-catenin, confirming the stroke-induced neurogenesis pathway. Ngb overexpression further increased the nuclei localization of β-catenin. These findings suggest that Ngb overexpression might promote neurogenesis at least in part through Wnt signaling. However, we did not investigate how Ngb overexpression may upreuglate Dvl1 expression and nuclear translocalization of β-catenine, which will be interesting subjects for future studies.

The proliferation and differentiation are generally considered opposite processes during development for a same group of stem cells. There are multiple studies showing one molecule or pathway that increases proliferation but inhibit differentiation, or vice versa. For example, overexpression of Dyrk1A, a member of Dyrk (dual-specificity tyrosine-(Y)-phosphorylation regulated kinase) family, has been reported to inhibit proliferation, but induce neuronal differentiation of neural progenitor cells^51^. On the other hand, if proliferation or differentiation has been initiated, some molecules/pathways may be able to affect proliferation and differentiation toward the same direction. This notion has been validated by a large group of experimental studies. For example, Esfandiari et al reported that inhibition of GSK3 (Glycogen synthase kinase 3) promotes both proliferation and neuronal differentiation of human neural progenitor cells^52^. Moreover, Neudesin^53^, Runx1^54^, and lithium^55^ have been reported to promote both proliferation and neuronal differentiation of neural progenitor cells. Here we studied the effect Ngb overexpression on proliferation and differentiation of culture NPCs when the cells were cultured either in proliferation or differentiation conditions, respectively, it is thus acceptable that Ngb overexpression can promote both proliferation and neuronal differentiation of neural progenitor cells.

Neurogenesis following stroke has been demonstrated to be a series of sequential events including neural progenitor cell proliferation, neuronal differentiation, maturation and migration towards lesion areas^56^. Experimental studies show that after BrdU injection in mice brain following stroke, the BrdU labeled cells are first Dcx (immature neuron marker) immunoreactive for a few weeks, then gradually lose this expression and become NeuN (mature neuron marker) immunoreactive^45,56^. It is therefore reasonable to speculate that the increased number of neuroblasts (PSA-NCAM positive) at 7 days post stroke in this study might result in an increased number of nearly-mature neurons (Tuj1 positive) at 14 days after stroke, and may eventually contribute to long term improved outcomes. Future studies may also be interesting to investigate whether Ngb affects the migration of new-born neurons towards lesion area, and whether they can integrate with existing neural circuits.

Nevertheless, there are a few caveats in this study. First, we did not use “loss-of-function” approach to define the roles of Ngb in neurogenesis, which will be planned in future studies using either siRNA or gene knockout approaches. Second, we tested the roles of Ngb overexpression in neurogenesis in ischemic stroke as an example of pathological conditions, but we did not test the functional outcomes after stroke. Additionally, whether Ngb also regulates neurogenesis in other conditions such as brain trauma or hemorrhagic stroke were not examined. These issues require careful investigation in the future. Third, in this study we only examined the roles of Wnt signaling in Ngb function in neurogenesis. As neurogenesis is a complex physiological process, multiple pathways other than Wnt signaling might also be involved, such as Notch1^57^, Sonic Hedgehog^58^, bone morphogenetic protein (BMP)^59^ and microRNA^60^ etc. Whether these pathways are also involved in the pro-neurogenesis effect of Ngb warrants further investigations in in the future.

Overall, in this study we for the first time demonstrated that Ngb overexpression promotes both proliferation and neuronal differentiation of cultured NPCs, and promotes neurogenesis in mice after stroke, potentially through Wnt signaling pathway. As endogenous Ngb upregulation may be a more promising strategy for stroke treatment^47,61^, these findings will broaden the spectrum of the neuroprotection mechanisms of Ngb in stroke. Since we recently have established a cell based screening system, and identified a few natural compounds that upregulate endogenous Ngb expression, such as formononetin^38^, in future studies we may use these Ngb-upregulating compounds to treat animals at either acute phase of stroke to confer neuroprotection, or at recovery phase to promote neurogenesis. These investigations may eventually benefit the development of therapeutic strategies targeting neurogenesis for neurological disorders such as stroke.

## Electronic supplementary material

Supplementary figure legends
