# Author Correction: Neuroglobin promotes neurogenesis through Wnt signaling pathway

**DOI:** 10.1038/s41419-019-1421-8

**Published:** 2019-03-01

**Authors:** Zhanyang Yu, Chongjie Cheng, Yu Liu, Ning Liu, Eng H. Lo, Xiaoying Wang

**Affiliations:** 1 Neuroprotection Research Laboratory, Departments of Neurology and Radiology, Massachusetts General Hospital, Harvard Medical School, Charlestown, MA USA; 2 Department of Neurosurgery, The First Affiliated Hospital of Chongqing Medical University, Chongqing, China; 3 Department of Neurology, the Second Affiliated Hospital, Harbin Medical University, Harbin, Heilongjiang China; 4 College of Medicine, Hunan Normal University, Changsha, China

**Correction to:** **Cell Death and Disease** 9: 945;

10.1038/s41419-018-1007-x;

published online 20 September 2018

The original version of this Article contained an error in the Abstract. “PSA-NCAM positive neuroblastoma cells” should have read “PSA-NCAM positive neuroblasts”. Similarly, in the first paragraph of the Discussion, “the number of neuroblastoma cells” should have read “the number of neuroblasts”.

This has been corrected in both the PDF and HTML versions of the Article.

