# Supplementary material for: Neuroglobin promotes neurogenesis through Wnt signaling pathway
Source: Cell Death Dis. 2018 Sep 20;9(10):945. doi: 10.1038/s41419-018-1007-x (PMC6147998; doi:10.1038/s41419-018-1007-x)
Supplement: Supplementary file 1 — Supplementary figure legends [file 41419_2018_1007_MOESM1_ESM.doc]

**Figure S1: Lv-GFP transduction in mice brain.**

Lv-GFP was administered through ICV injection to confirm the efficiency of lentivirus transduction in mice brain. The mice was sacrificed at 3 days after ICV injection and sectioned for GFP fluorescence imaging. (A) Sham group; (B) Lv-GFP injection group.
